# Chronic nicotine reverses age-related decline of interneuron markers in mouse auditory cortex

**DOI:** 10.3389/fnagi.2026.1876409

**Published:** 2026-09-09

**Authors:** Irakli Intskirveli, Ronit Lazar, Dianna Hidalgo, Raju Metherate

**Affiliations:** Department of Neurobiology and Behavior, Center for Hearing Research, University of California, Irvine, Irvine, CA, United States

**Keywords:** auditory cortex, CHRNA2, chronic nicotine exposure, hearing, interneuron, mouse, nicotinic acetylcholine receptor

## Abstract

Inhibition in central auditory pathways declines in old age, which contributes to age-related auditory processing deficits. In auditory cortex, most inhibitory interneurons can be identified by one of three marker proteins: parvalbumin (PV), somatostatin (SOM), or vasoactive intestinal peptide (VIP). About 10% of interneurons contain the α2 nicotinic acetylcholine receptor (α2 cells), and most of these are layer 5 Martinotti cells (α2-MCs). We examined the distribution of these four interneuron subtypes in auditory cortex of FVB mice, from young adult through old age, and the extent to which α2 cells express the marker proteins. We also examined the effects of chronic nicotine exposure (CNE) at older ages, as a step toward potential therapeutic applications. We found that the numbers of cells expressing PV, SOM and VIP, and α2-MCs, all declined with age, but total α2 cells did not. We also found that most (86%) α2 cells in young adult mice co-expressed SOM, but the co-expression declined sharply with age. Notably, CNE for three months—starting at age 9, 15, or 21 months—increased the numbers of cells with PV and VIP at 12, 18, and 24 months, respectively, to levels seen in young adults. CNE increased SOM interneurons only at 12 months and had no effect on the number of α2 cells. An analysis of immunofluorescence intensity in 12-month-old mice suggested that the increase in cell counts after CNE resulted from increased expression of marker protein, rather than *de novo* appearance of interneurons. Thus, CNE partly or fully reversed the age-related decline of interneuron-marker expression and may prove useful for treatment of age-related auditory processing deficits.

## 1 Introduction

The ability to process auditory stimuli, notably speech, declines in old age due to combination of peripheral and central deficits (Dalton et al., 2003; Gordon-Salant et al., 2006; Weinstein and Ventry, 1982). Treatments for peripheral deficits, such as hearing aids, improve audibility but do not address central deficits. Central inhibition is important for auditory processing, but inhibition declines in old age independently of peripheral hearing loss (Brewton et al., 2016; Caspary et al., 2008; Rogalla and Hildebrandt, 2020; Turner et al., 2005). Ideally, treatments for age-related processing deficits should compensate for central, as well as peripheral, deficits. For example, drugs that target central inhibition could be used in combination with hearing aids to reverse the age-related decline in auditory processing.

In cortex, inhibitory interneurons are found mostly (>80%) in three non-overlapping groups identified by the marker proteins parvalbumin (PV), somatostatin (SOM), or vasoactive intestinal peptide (VIP) (Rudy et al., 2010). Some interneurons in auditory cortex exhibit age-related decline (i.e., decline in the number of cells with, and/or intensity of, marker-protein expression), although there are species and strain differences (see sections “4 Discussion, 4.1 Laminar profile and age-related decline of interneuron markers in auditory cortex”) (Brewton et al., 2016; Ouda et al., 2008; Ouellet and de Villers-Sidani, 2014; Rogalla and Hildebrandt, 2020). A potential pharmacological approach to compensate for loss of cortical inhibition is to increase activation of nicotinic acetylcholine receptors (nAChRs) that can mediate excitation of interneurons (Askew et al., 2019; Jia et al., 2009; Jones and Yakel, 1997). Notably, systemic administration of nicotine alters spectral integration in rodent auditory cortex to “sharpen” frequency receptive fields—producing narrower receptive fields with increased gain (Askew et al., 2017; Intskirveli et al., 2024; Intskirveli and Metherate, 2012; Liang et al., 2008)—which mimics the effect of auditory attention in humans and monkeys (Lakatos et al., 2013; O’Connell et al., 2014; Okamoto et al., 2007). Potentially, this sharpening effect of nicotine on cortical receptive fields could offset the age-related broadening of receptive fields associated with reduced inhibition (Bishop et al., 2022; Turner et al., 2005). These results suggest that acute or chronic nicotine may be useful for treatment of age- and attention-related auditory processing deficits. Indeed, systemic nicotine can reverse the age-related decline of some measures of auditory processing in older mice and humans (Hussain et al., 2026; Rumschlag et al., 2022; Sun et al., 2021), and chronic exposure can improve cognitive function in older humans (Majdi et al., 2021; Newhouse et al., 2012). The mechanisms by which nicotine may enhance function in the aging brain remain unclear.

In cortex, 90% of nAChRs are of the α4β2 subtype while the remainder are mostly α7 (Mao et al., 2008), and these nAChRs decline with age (Araujo et al., 1990; Ghimire et al., 2020; Sottile et al., 2017; Whitehouse et al., 1988; Zhang et al., 1990). The α2 nAChR is relatively scarce, comprising less than 3% of cortical nAChRs in mouse (Mao et al., 2008), but is increasingly implicated in important cortical and cognitive functions (Hilscher et al., 2017; Hilscher et al., 2023; Lotfipour et al., 2013; Lotfipour et al., 2017; Mineur et al., 2020; Siwani et al., 2018). In cortex, neurons with α2 nAChRs (here referred to as α2 cells) are inhibitory interneurons and mostly layer 5 Martinotti cells (α2-MCs) (Hilscher et al., 2017; Hilscher et al., 2023; Hostetler et al., 2023). The α2-MCs send a primary axon to superficial layers of the cortex to directly inhibit distal apical dendrites of layer 5 pyramidal neurons, and indirectly disinhibit (via inhibition of superficial interneurons) their proximal apical dendrites (Donato et al., 2023). This circuitry is hypothesized to mediate nicotinic sharpening of receptive fields in auditory cortex (Intskirveli et al., 2024). It is not known how α2 cells are affected by aging.

To better understand the interactions of systemic nicotine, α2 cells and inhibitory processes, here we examine in auditory cortex of FVB mice the distribution of four interneuron groups—PV, SOM, VIP and α2 cells—including their laminar profiles and changes with age, co-labeling of α2 cells with the other markers, and the effect of chronic nicotine exposure (CNE) at three older ages. Interneuron counts were obtained throughout the adult lifespan (2–24 months) using immunohistochemistry in transgenic mice that express enhanced green fluorescent protein (EGFP) in α2 cells. We found that while PV, SOM and VIP neurons, and α2-MCs, all declined with age, total α2 cells did not. Notably, 3-month CNE increased the numbers of PV and VIP interneurons at 12, 18, or 24 months to levels near those seen in young adults, and an analysis of PV immunofluorescence intensity in 12-month-old mice suggested that CNE increased expression of PV protein. CNE increased SOM numbers only at 12 months and had no effect on α2 cells. Thus, CNE can partly or fully reverse the age-related decline of interneuron marker expression in auditory cortex and may support treatment of age-related auditory processing deficits.

## 2 Materials and methods

### 2.1 Animals

We used transgenic mice that expressed EGFP in cells with α2 nAChRs (Chrna2 gene). The mouse line (Chrna2-EGFP) was obtained cryopreserved from the Mutant Mouse Resource and Research Centers (RRID: MMRRC 036130-UCD), reanimated on an FVB background by the UC Irvine Transgenic Mouse Facility, and bred with wild-type FVB mice. Mutant and wild-type offspring were identified by genotyping and only mutant mice were used in this study. Male and female mice were housed separately, five animals per cage, on a 12:12 h light-dark cycle. Mice of 9-, 15-, or 21-months age were randomly assigned to a control or CNE group and housed in pairs. Anatomical studies were conducted on mice of age 2, 12, 18, or 24 months. All procedures were in accordance with the National Institutes of Health Guide for the Care and Use of Laboratory Animals and were approved by the University of California, Irvine, Institutional Animal Care and Use Committee.

### 2.2 Chronic nicotine exposure

3-month CNE was via nicotine in drinking water (70 μg/ml free base, from nicotine hydrogen tartrate; Glentham Life Science) with 2% saccharine to mask the taste (Rogers et al., 1998). Littermate control mice received saccharine only. CNE mice were adapted to nicotine gradually with 9 μg/ml on day one, 17 μg/ml on days 2–3, 35 μg/ml on days 4–6, and 70 μg/ml thereafter (Rogers et al., 1998). Mice were visually assessed daily for overall health. Drinking water was replenished once a week, at which time mice were weighed to confirm maintained weight.

In preliminary experiments, a subset of CNE mice was tested for blood levels of cotinine, a stable nicotine metabolite that is an established biomarker of exposure (Benowitz, 1996). Blood samples obtained from the facial vein (Stewart and Schroeder, 2017) were analyzed by the University of California, San Francisco, Clinical Pharmacology Laboratory. Cotinine levels averaged 42 ± 7.7 ng/ml (*n* = 4) in mice subject to 3-month CNE, and 36 ± 6.9 ng/ml (*n* = 3) in mice subject to 6-month CNE. The two groups were not different (unpaired *t*-test, *t* = 0.5742, *p* = 0.5907), suggesting that cotinine levels had plateaued with 3-month exposure. Control mice (saccharine only, 6-month exposure, *n* = 2) did not have measurable levels of cotinine (<10 ng/ml). These results demonstrate the effectiveness of the 3-month CNE method of delivery.

### 2.3 Immunohistochemistry

Mice were deeply anesthetized with urethane (0.7 g/kg, i.p., Sigma) and xylazine (13 mg/kg, Phoenix Pharmaceutics), perfused transcardially with phosphate-buffered saline (PBS, 0.1 M sodium phosphate, 0.9% NaCl) followed by 4% paraformaldehyde (PFA) for 10 min using a peristaltic pump (10 ml/min). Brains were fixed overnight in 4% PFA at 4 deg C. After rinsing with PBS, the brain was sectioned along the plane of the auditory thalamocortical projection (Cruikshank et al., 2002) on a microtome (VT1000P, Leica Biosystems). We sectioned brains starting from the ventral surface and began saving 30 μm-thick sections after 2715 ± 24 μm, with the appearance of the expected anatomical structures including clearly identified medial geniculate body, lateral geniculate nucleus, fimbria, hippocampus, and cortex (Cruikshank et al., 2002). From that point, sectioning dorsally over ∼270 μm, we obtained nine 30 μm sections to create three sets of sections, with each set immuno-stained for SOM, VIP and PV (one antibody per section). PV sections were counterstained with DAPI to identify cell nuclei.

Sections were rinsed in PBS and incubated for 90 min in a blocking solution containing 5% donkey serum and 0.3% Triton X-100 in PBS (pH 7.4) at room temperature. Sections were then placed in a solution containing primary antibodies at 4 deg C for 24 h: anti-PV (monoclonal antibody from mouse, clone PARV-19; dilution 1:1000; catalog no. P3088-100UL, Millipore Sigma), anti-SOM (monoclonal antibody from rat, dilution 1:500; catalog no. MAB354, Millipore Sigma), or anti-VIP (monoclonal antibody from mouse, dilution 1:500; catalog no. SC-2534, Santa Cruz Biotechnology). Then, sections were rinsed three times for 10 min each in PBS and secondary antibodies added for PV and VIP (donkey anti-mouse IgG, Alexa Fluor Plus 594, 1:500 dilution, catalog no. PIA32744, Invitrogen) or SOM (donkey anti-rat IgG, Alexa Fluor 594, 1:500 dilution, catalog no. A48271, Invitrogen) in blocking solution for 120 min at room temperature in the dark. Afterward, sections were washed three times for 10 min each in PBS. For SOM and VIP staining, to reduce background staining in mice 12 months and older, floating sections were then incubated with 0.3% Sudan Black B (Lipofuscin Autofluorescence Quencher, Thermo Scientific) in 70% Ethanol for 3 min at room temperature and washed three times for 5 min in water (Oliveira et al., 2010). Tests in 2-month-old mice, *n* = 4, showed that Sudan Black B treatment had no effect on α2 cell counts. Sections stained for PV were incubated for 10 min in 10 μM DAPI (Sigma) in PBS and washed three times for 10 min in PBS. All sections were mounted on slides with Glycergel mounting medium (Dako).

### 2.4 Imaging and cell counting

We used a microscope (Axioskop A1, Zeiss) equipped with a digital camera (AxioCam HRc) and fluorescent light source (X-Cite; 120 Q Series, EXFO Photonic Solution), with imaging software to acquire and analyze images (Zen 2, Blue addition, Zeiss). To define a region of interest for counting cells in auditory cortex, we relied on our experience with anatomical procedures in auditory thalamocortical sections after physiological verification *in vivo* (tonotopic organization) or *in vitro* (lemniscal auditory thalamocortical transmission) (Askew et al., 2019; Cruikshank et al., 2001; Cruikshank et al., 2002; Intskirveli et al., 2024; Intskirveli and Metherate, 2012; Kawai et al., 2007). We first drew a reference line orthogonal to the cortical surface from the tip of the hippocampus where the fimbria emerges. This reference likely runs through primary auditory cortex. We then drew two parallel lines ± 500 μm to the reference in anterior and posterior directions, to define a region of interest also bounded by the cortical surface and the border between layer 6 and subcortical white matter. To define cortical layers, the cortical width of each section was measured at three places: along the reference line and the anterior and posterior edges of the region of interest. These measurements were used to subdivide the cortex into layers, per Anderson et al. (2009). Thus, the ∼1 mm^2^ region of interest for cell counting was divided into six sub-regions that comprised anterior and posterior divisions of three laminar groups: supragranular (layers 1–3; 38% of cortical width), granular (layer 4; 12% of cortical width), and infragranular (layers 5–6; 50% of cortical width). Cells were counted in each sub-region and the results used for statistical analyses.

Cell counts for α2 cells (EGFP) and for interneurons with PV, SOM and VIP (immunofluorescence) were obtained from 20× images by manual counting (using Zen 2 software), and this manual method was validated in a subset of sections using a semi-automated procedure. For manual counting, in images taken at a single plane of focus we counted clearly delineated cell bodies, typically with one or more processes extending from the soma. The threshold fluorescence for counting a cell was approximately 3 standard deviations above background levels. Counting was performed independently by three investigators. Since we had no *a priori* hypothesis for the effects of CNE, counting was not explicitly blind but images were not labeled with the experimental condition and CNE effects were not evaluated until final analysis of data from all ages and conditions (over 4000 images). To validate this manual approach, we re-counted cells in 12-month-old mice using an automated procedure with Polygon AI software (Rewire AI, Inc) (Slaker et al., 2016) followed by manual review of each image by an investigator who was blinded to the experimental condition and different from the investigator who performed the manual counting. Polygon AI software was trained using a set of five tissue sections and a biomarker-specific detection model (Rewire AI) and then used to automatically analyze the remaining 12-month data (∼70 sections from 26 mice). Each image was then reviewed by the blinded investigator to confirm that automated counting included only cells and not artifacts. Polygon AI output included fluorescence intensity measures for each cell, with background subtracted (Slaker et al., 2016).

### 2.5 Statistical analyses

Statistical analyses were performed with GraphPad PRISM v9.1.2. Mean data are reported ± SE. To test the difference between two or more independent groups, we used an unpaired *t*-test or one-way ANOVA (α = 0.05). A two-way ANOVA (α = 0.05), followed by *post hoc* Tukey’s test, was used to determine main effects for each of two independent variables plus interaction effects. In the Results for each interneuron subtype, data for each mouse are the mean number of cells per tissue section, which is the average of three sections, with each mouse contributing one value toward the reported mean (i.e., “n” indicates the number of mice).

## 3 Results

**FIGURE 1:**
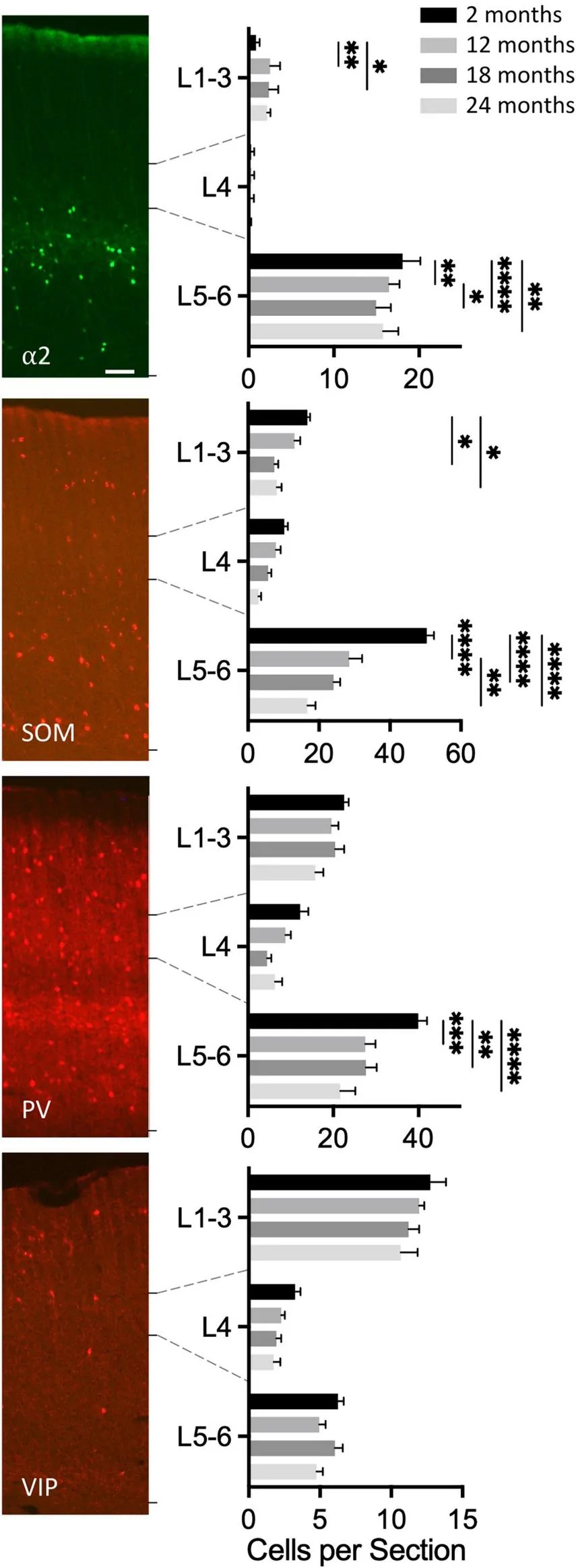
Laminar distribution of interneuron subtypes over 2–24 months age (asterisks reflect *post hoc* within-layer comparisons: *p < 0.05; **p < 0.002; ***p < 0.0002; ****p < 0.0001). Scale bar indicates 100 μm. Number of mice at each age: 2 months, *n* = 7–11; 12 months, *n* = 11–16; 18 months, *n* = 6–8; 24 months, *n* = 4–5.

We visualized fluorescent antibodies against PV, SOM and VIP in brain sections obtained from 65 transgenic mice expressing EGFP in cells with α2 nAChRs (Chrna2-EGFP mice). Cells with fluorescent antibodies indicating the presence of marker proteins are referred to as “PV interneurons”, etc. Brains were obtained at four ages, from 2 to 24 months, spanning the adult lifespan of FVB mice (Yuan et al., 2009), and sections were cut along the plane of the auditory thalamocortical projection (Cruikshank et al., 2002) in order to target auditory cortex. Within a 1 mm^2^ region of interest centered in auditory cortex, cells were counted in three groups: supragranular (layers 1–3), granular (layer 4), and infragranular (layers 5–6) (Figure 1). Each group initially was sub-divided into equal anterior and posterior regions that, given the near-horizontal slice plane, roughly correspond to higher and lower frequency representations. The anterior and posterior subdivisions were combined after demonstration of no differences in cell counts across ages (2-way ANOVAs, *p* > 0.05). Similarly, cell counts across sections over the dorsoventral extent of auditory cortex (three sets of 30 μm-thick tissue sections spanning ∼270 μm) also showed no age-related differences (2-way ANOVAs, *p* > 0.05) and were combined. Thus, for each interneuron subtype, results for each mouse consist of the mean number of cells per tissue section averaged across anterior-posterior and dorsal-ventral dimensions. In the Results that follow, 2-month data are from untreated mice, whereas older mice were CNE-treated (nicotine plus saccharine in drinking water) or littermate controls (saccharine only); however, 12-month controls include both untreated mice and saccharine-only controls that were combined due to no difference (unpaired *t*-tests with Welch’s correction, *p* > 0.05). The number of mice at each age are (M, male; F, female): 2 months: 6M, 5F; 12 months: control 10M, 6F; CNE 6M, 4F; 18 months: control 2M, 6F; CNE 2M, 6F; 24 months: control 5F, CNE 7F. The reduced numbers at 24 months reflect loss of male mice since female FVB mice exhibit a longer lifespan (Yuan et al., 2009).

### 3.1 Laminar distributions change with age

Figure 1 shows fluorescent images for each interneuron subtype in young adult, 2-month-old mice (green EGFP for α2 cells; red immunohistochemistry for interneuron marker proteins), with histograms showing cell counts for each laminar group across the four ages. For each interneuron, data were analyzed by 2-way ANOVA and the results reported below. Differences across layers (laminar profile) are reported, as are age-related changes and any interaction (i.e., change in laminar profile with age). Age-related changes *within* layers are shown in Figure 1 (asterisks, Tukey *post hoc* tests). Except as noted, laminar profiles did not change with age; thus, in subsequent figures, data are combined across layers.

**FIGURE 2:**
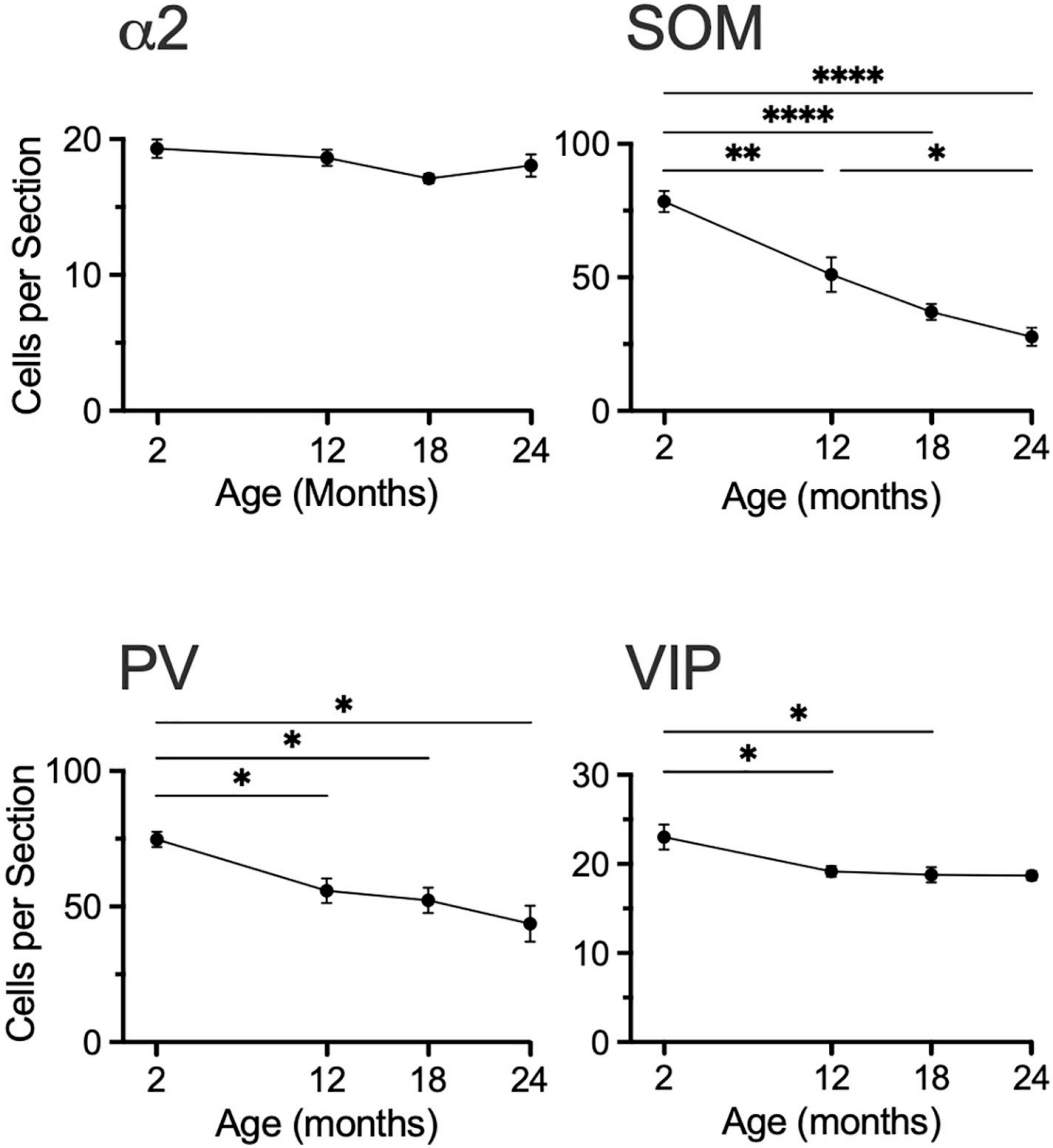
Age-related changes in interneuron counts. Asterisks for *post hoc* results and number of mice at each age are the same as in Figure 1.

Neurons with α2 nAChRs—referred to as α2 cells, or for infragranular cells, α2-MCs—show a clear laminar difference (Figure 1, α2) (F (2, 99) = 1867, *p* < 0.0001) with the large majority being infragranular, about 10% supragranular, and almost none in layer 4. While overall there was no change with age (F (3, 99) = 1.568, *p* = 0.2019) (cf. Figure 2), an age-related increase in supragranular cells and decrease in infragranular cells produced a small change in the laminar profile (interaction F (6, 99) = 7.160, *p* < 0.0001). Note that the band of fluorescent label in layer 1 (Figure 1, α2), includes processes (presumably axon terminations of α2-MCs) but no cell bodies, and the small number of supragranular α2 cells were typically in layer 2/3.

SOM interneurons were found in all layers, with greater numbers in deeper layers (Figure 1, SOM) (F (2, 90) = 125.7, *p* < 0.0001). There is a progressive decrease with age (F (3, 90) = 35.23, *p* < 0.0001) that is most prominent in deep layers, leading to an age-related change in laminar profile (interaction F (6, 90) = 10.44, *p* < 0.0001).

PV interneurons were found in all layers with a small laminar difference (Figure 1, PV) (F (2, 90) = 76.74, *p* < 0.0001), though with similar cell density across layers when accounting for the different area of each laminar group (infragranular area > supragranular > granular). PV cell numbers decreased with age (F (3, 90) = 8.623, *p* < 0.0001), including a prominent decrease for the infragranular group, but similar trends occurred in each lamina and there was little change in the laminar profile with age (interaction, F (6, 90) = 1.816, *p* = 0.1047).

VIP interneurons were found in all layers, but with greater numbers in the supragranular group (Figure 1, VIP) (F (2, 81) = 205.7, *p* < 0.0001). There was a weak overall decrease with age (F (3, 81) = 3.600, *p* = 0.0169) (cf. Figure 2), but no age-related changes within layers and no change in laminar profile (interaction F (6, 81) = 0.4732, *p* = 0.8264).

### 3.2 Age- and sex-related changes in interneuron counts

For each interneuron subtype, Figure 2 shows total counts (combined across laminae) at each age. Across 2–18 months, groups include both sexes and results were analyzed by 2-way ANOVA (as noted above, no male mice survived to 24 months). With only minor sex differences observed, as reported below, data were combined across sex and analyzed over 2–24 months for the results in Figure 2 (asterisks indicate results of *post hoc* tests).

The number of α2 cells did not change over 2–24 months (Figure 2, α2) (F (3, 34) = 1.647, *p* = 0.1968) and no sex differences were observed (F (1, 27) = 2.096, *p* = 0.1592). In contrast, SOM interneurons exhibited a strong and progressive decline with age (Figure 2, SOM) (F (3, 31) = 15.92, *p* < 0.0001), with no sex difference (F (1, 24) = 0.00086, *p* = 0.9768). PV interneurons declined with age (Figure 2, PV) (F (3, 30) = 4.481, *p* = 0.0103) with much of the change occurring at 12 months; again, there was no sex difference (F (1, 26) = 0.8849, *p* = 0.355). VIP interneurons also decreased with age, primarily at 12 months (Figure 2, VIP) (F (3, 27) = 3.406, *p* = 0.0318) and, only at 2 months, exhibited a greater number of cells in male mice (F (1, 24) = 6.450, *p* = 0.0180). Thus, the numbers of SOM, PV and VIP neurons all declined with age, with most changes occurring by 12 months, but the number of α2 cells did not change over the adult lifespan.

### 3.3 Co-labeling of α2 cells with interneuron markers

**FIGURE 3:**
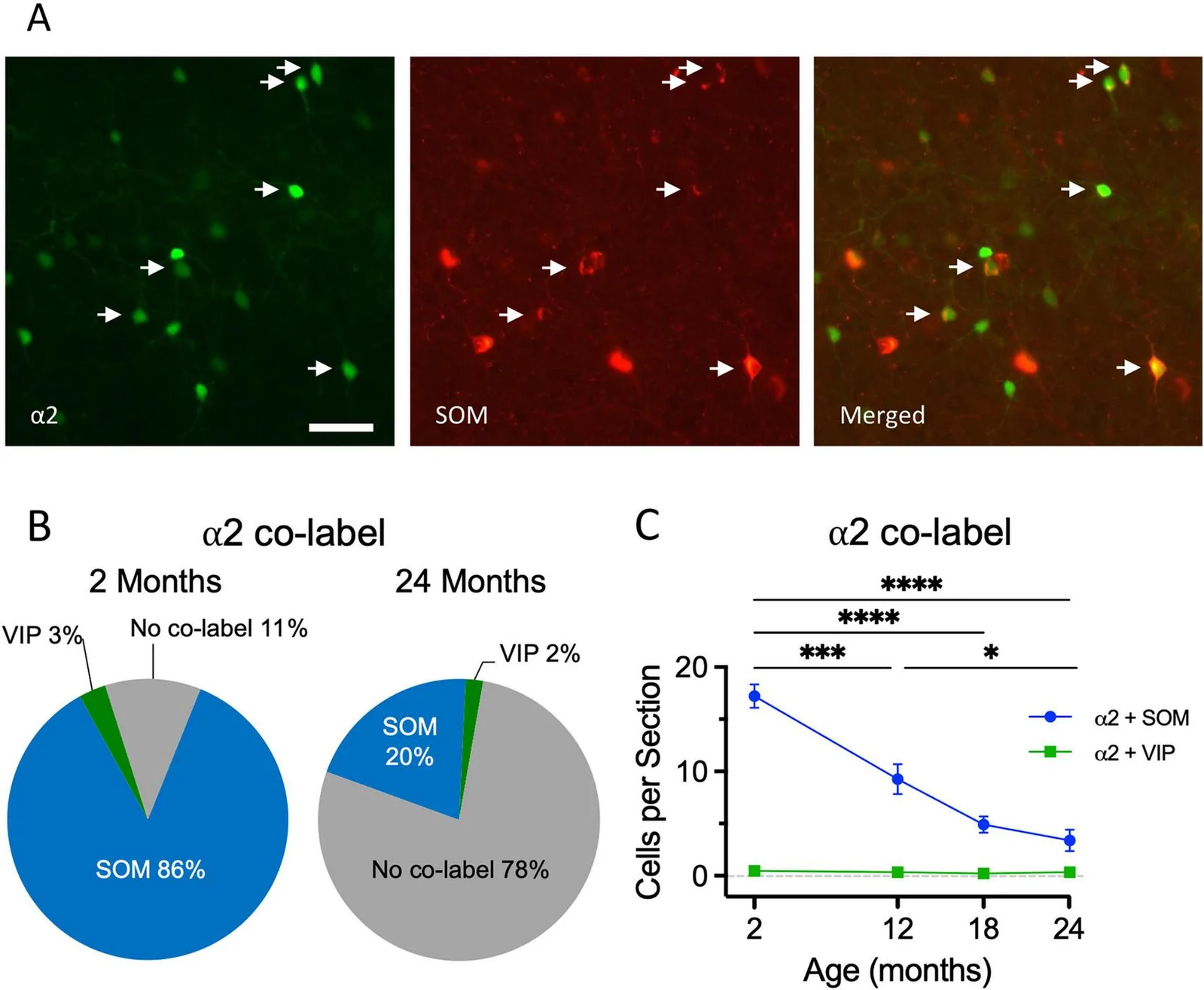
Co-labeled α2 cells. **(A)** Example of infragranular cells from a 2-month-old mouse (arrows indicate cells co-labeled with SOM; scale bar, 50 μm). **(B)** Proportion of α2 cells that were co-labeled at 2 and 24 months. **(C)** Number of α2 cells co-labeled with SOM (asterisks for *post hoc* results as in Figure 1) or VIP (no change with age). Number of mice at each age as in Figure 1.

Figure 3 shows the extent to which α2 cells were co-labeled with other interneuron markers. In 2-month-old mice, 85.9% of α2 cells were co-labeled with SOM and few (3.1%) with VIP; none were co-labeled with PV (Figures 3A, B). The remaining 11.0% of α2 cells were otherwise unidentified. Most α2 cells co-labeled with SOM were infragranular (e.g., Figure 3A), whereas the few co-labeled with VIP were supragranular. Notably, the α2 cells co-labeled with SOM decreased progressively with age, from 86% at 2 months to 20% at 24 months (Figures 3B,C) (1-way ANOVA, F (3, 30) = 20.35, *p* < 0.0001), but the small number co-labeled with VIP remained unchanged (F (3, 28) = 0.5101, *p* = 0.6785).

### 3.4 Effect of chronic nicotine exposure on age-related decline of interneurons

**FIGURE 4:**
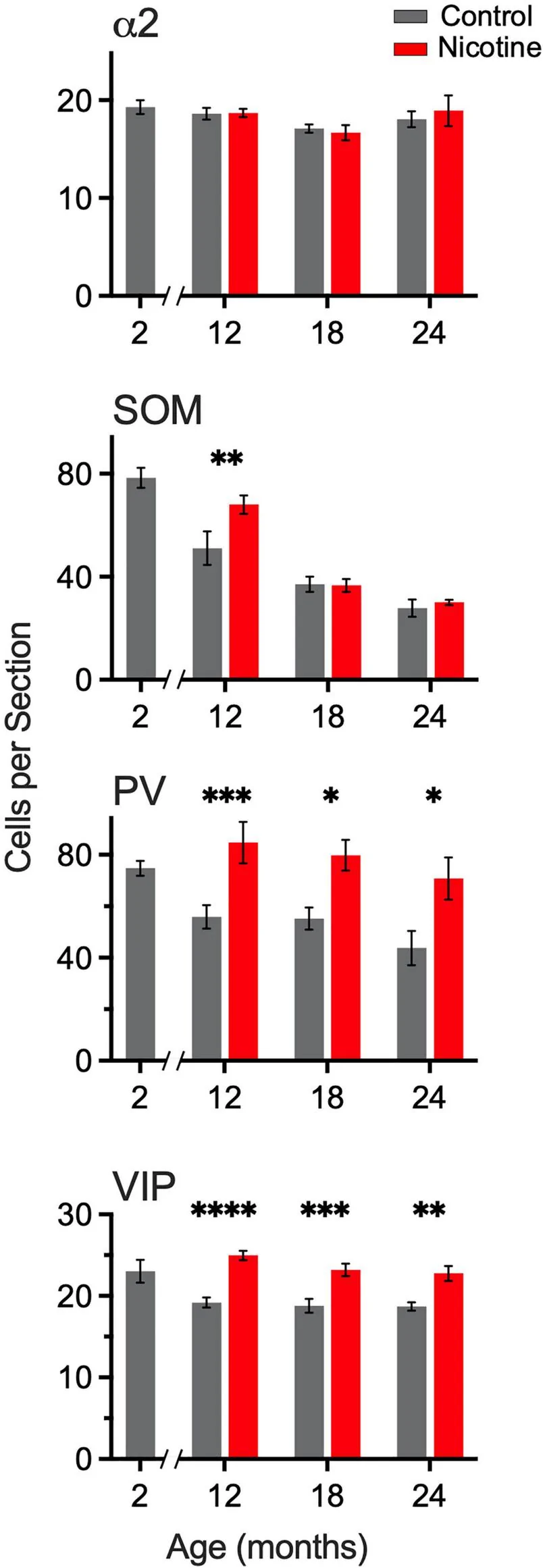
Effect of 3-month CNE on interneuron numbers. Results are from separate groups of mice tested at 12, 18 or 24 months. For *post hoc* comparison of CNE vs. controls at each age, asterisks are as in Figure 1. Number of mice at each age: 2 months, *n* = 7–11; 12 months: CNE *n* = 10, control *n* = 11–16; 18 months: CNE *n* = 8, control *n* = 6–8; 24 months: CNE *n* = 6–7, control *n* = 4–5.

CNE was tested in separate groups of mice at three ages, beginning at 9, 15, or 21 months and continuing for three months. CNE mice received nicotine in their drinking water, with saccharine to mask the taste, whereas littermate controls received saccharine alone. Mice were weighed weekly to confirm that CNE and control groups maintained their weight over the 3-month treatment (2-way repeated measures ANOVAs, *p* > 0.05). CNE effects on cell counts were compared to controls at 12, 18, and 24 months (2-way ANOVA). The results are shown in Figure 4, which also includes data from untreated mice at 2 months for comparison (control data are the same as in Figure 2, but asterisks in Figure 4 refer to the outcome of *post hoc* tests for CNE vs. control).

CNE had no effect on the number of α2 cells (Figure 4, α2) (F (1, 46) = 0.0561, *p* = 0.8137) and increased the number of SOM cells at 12 months (Figure 4, SOM) (F (1, 41) = 22.36, *p* = 0.1386; *post hoc* test at 12 months, *p* < 0.0060). Notably, CNE increased PV and VIP cell counts at all three ages (Figure 4, PV, VIP) (PV: F (1, 45) = 20.41, *p* < 0.0001; VIP: F (1, 39) = 39.78, *p* < 0.0001). Thus, the effects of 3-month CNE largely opposed the age-related decline of interneuron markers over 12–24 months.

The effects of CNE on total cell counts (Figure 4) generally were not laminar-specific, i.e., similar effects occurred across cortical layers (not shown). For α2 cells, 2-way ANOVA followed by *post hoc* tests to examine supragranular and infragranular cells separately showed no CNE effect (supragranular: *p* = 0.6923; infragranular: *p* = 0.5422). For SOM cells, increased cell counts at 12 months occurred in each laminar group (supragranular: *p* < 0.0111; granular: *p* < 0.0027; infragranular: *p* < 0.0056). For PV cells, the increased cell counts at 12 and 18 months occurred in each laminar group (12 months, supragranular: *p* < 0.0159; granular: *p* < 0.0374; infragranular: *p* < 0.0006; 18 months, supragranular: *p* < 0.0284; granular: *p* < 0.0287; infragranular: *p* < 0.0259), but at 24 months the effects in individual layers showed trends that were not significant (*p* > 0.05). For VIP cells, the increased cell counts at 12 months occurred in each laminar group (supragranular: *p* < 0.0009; granular: *p* < 0.0161; infragranular: *p* < 0.0211), and at 18 and 24 months the increased counts occurred in supragranular layers (18 months: *p* < 0.0223; 24 months: *p* < 0.0095), without significant effects in granular and infragranular groups (*p* > 0.05). Thus, for the most part, CNE effects in each laminar group resembled the effects on total cells shown in Figure 4.

The effects of CNE did not differ by sex, except that the increase in SOM cells at 12 months was greater in male mice (2-way ANOVA with Tukey *post hoc*, *p* = 0.0086). We also examined the effect of CNE on the number of co-labeled cells (not shown). For α2 cells co-labeled with SOM, even as the number of co-labeled cells decreased with age (Figure 3C), there was no effect of CNE across ages (2-way ANOVA, F (1, 42) = 0.6752, *p* = 0.4159). For α2 cells co-labeled with VIP, which remained scarce across ages (Figure 3C), there was a trend toward increased numbers with CNE (F (1, 41) = 3.489, *p* = 0.0689) that attained significance only at 18 months (Tukey *post hoc* test, *p* = 0.0366).

**FIGURE 5:**
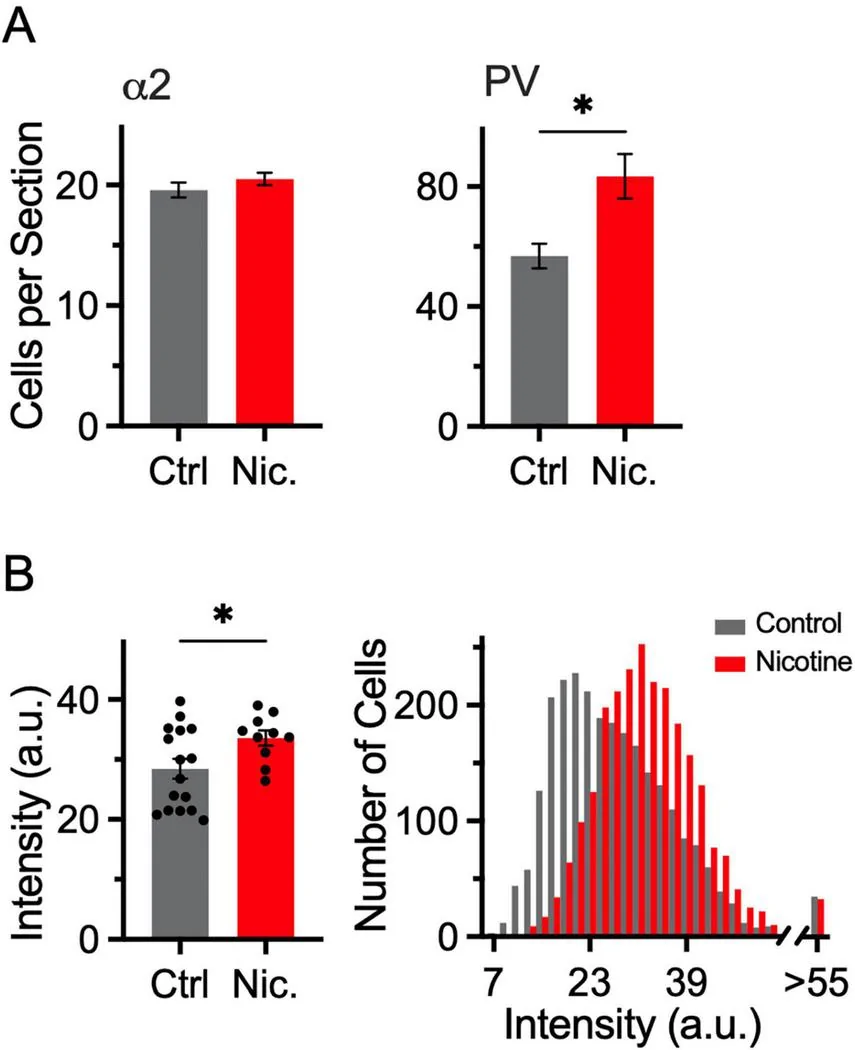
Effect of CNE on α2 and PV cell numbers **(A)** and PV immunofluorescence intensity **(B)** in 12-month-old mice. **(A)** α2 and PV cell numbers using semi-automated counting method. **(B)** Mean PV immunofluorescence intensity (left, data points are mean intensity per mouse; control *n* = 16, CNE *n* = 10; a.u., arbitrary units) and distribution of intensity for all PV cells (right; control *n* = 2565 cells; CNE *n* = 2429 cells; Mann Whitney U test, *p* < 0.0001). For statistical comparisons, asterisks are as in Figure 1 and number of mice are the same as in Figure 4.

For a subset of the data in Figure 4, we performed a *post hoc* validation of our manual counting method using a semi-automated procedure (automated counting with review by a blinded investigator, see Materials and Methods). Specifically, we re-analyzed the effect of CNE on α2 cells and PV interneurons in 12-month-old mice. The results confirmed that CNE had no effect on the number of α2 cells (Figure 5A, left; unpaired *t*-test with Welch’s correction, *t* = 1.138, *p* = 0.2665) and increased PV cell counts (Figure 5A, right; t = 3.161, *p* = 0.0067) with, in both cases, cell counts nearly identical to manual counts (cf. Figure 4). For additional insight into the effect of CNE, we examined the intensity of fluorescence in PV cells. CNE increased intensity, measured as the mean value per mouse (Figure 5B, left; *t* = 2.432, *p* = 0.0228). Since a visual comparison of individual data points in control and CNE groups (Figure 5B, left) appeared to show a preferential loss of weak-intensity fluorescence after CNE, we analyzed the distribution of fluorescence intensity for all cells. The results (Figure 5B, right) showed that CNE systematically shifted the distribution to higher intensities, with a 22% increase in median value (Mann Whitney U test, *p* < 0.0001). Note that for both the per-mouse means (Figure 5B, left) and intensity distributions for individual cells (Figure 5B, right), the shift to higher intensities occurred within the overall range observed in control mice, i.e., there was no increase in maximum value. Thus, the semi-automated analysis (Figure 5) validated our manual counting (Figure 4) and suggested that CNE increased expression of PV in 12-month-old mice without exceeding the maximum expression in control mice.

### 3.5 Age- and CNE-related changes in cortical width

Finally, for insight into broader changes in auditory cortex over the adult lifespan of FVB mice, we examined overall cortical width—the distance from the pia through layer 6—over ages 2–24 months. Cortical width averaged 1078 ± 9.85 μm at 2 months and decreased 13% with age (1-way ANOVA, F (3, 34) = 37.50, *p* < 0.0001), with the entire decrease seen at 12 months (Tukey *post hoc*, *p* < 0.0001). There was no sex difference in cortical width across ages (2-way ANOVA, F (1, 28) = 0.1516, *p* = 0.700). Moreover, CNE did not affect cortical width (2-way ANOVA, F (1, 42) = 1.783, *p* = 0.1890). Thus, while early decreases in interneuron numbers (Figure 2, 12-month data) were associated with decreased cortical width, similarly in both sexes, later interneuron decreases as well as the increased numbers following CNE were not associated with changes in overall cortical width.

## 4 Discussion

We examined age- and CNE-related changes in the distribution of interneurons in auditory cortex of mice that express EGFP in neurons with α2 nAChRs. The α2 cells were mostly infragranular (presumed α2-MCs) and comprised ∼10% of the interneuron population (Figure 1). The numbers of interneurons with PV, SOM and VIP declined over the adult FVB lifespan (2–24 months), with most changes occurring by 12 months, but the number of α2 cells did not change with age (Figure 2). Most (86%) α2 cells in 2-month-old mice were SOM interneurons, but this co-labeling decreased sharply to 20% in 24-month mice (Figure 3). CNE increased the numbers of PV and VIP interneurons at older ages to levels seen in young adults, and increased SOM numbers at 12 months, but had no effect on α2 cells (Figure 4). An analysis of immunofluorescence intensity in 12-month-old mice suggested that the increase in PV cell counts after CNE resulted from increased expression of PV protein (Figure 5), rather than *de novo* appearance of PV interneurons. Overall, CNE partly or fully reversed the age-related decline of interneuron markers in auditory cortex.

### 4.1 Laminar profile and age-related decline of interneuron markers in auditory cortex

The PV, VIP, and SOM marker proteins identify > 80% of cortical interneurons, with the remainder being non-VIP 5-HT3A receptor-expressing interneurons (Rudy et al., 2010). Their laminar distributions and age-related changes reported here are broadly consistent with prior descriptions for rat and mouse auditory cortex, although there can be significant differences even between strains of the same species (Brewton et al., 2016; Ouda et al., 2008; Ouellet and de Villers-Sidani, 2014; Reinhard et al., 2019; Rogalla and Hildebrandt, 2020; Rudy et al., 2010). For example, PV interneurons decline with age in Fisher 344 rats and C57BL/6 mice (and FVB mice, present study), but not in Long-Evans rats or CBA/CaJ mice (Brewton et al., 2016; Ouda et al., 2008). The decline of PV interneurons is thought to be independent of age-related peripheral hearing loss (Brewton et al., 2016; Rogalla and Hildebrandt, 2020), which nonetheless is less prominent in FVB mice than in other mouse strains (Ho et al., 2014; Rumschlag and Razak, 2021; Zheng et al., 1999). In our study, all three interneuron subtypes were distributed across cortical layers, and all declined with age. Most of the decline occurred by 12 months, although SOM interneurons continued to decline through 24 months. Only minor sex differences were observed, although it should be noted that only female mice survived to the oldest age (24 months), and sex differences were not tested at that age. Similarly, overall cortical width decreased 13% with age, with most of the change occurring by 12 months. The decline of interneuron markers is consistent with a general age-related loss of inhibitory function in central auditory pathways (Caspary et al., 2008; Shilling-Scrivo et al., 2021; Turner et al., 2005).

### 4.2 α2 nAChRs and α2-Martinotti cells

Among nAChR subtypes, α2 nAChRs are relatively scarce—comprising < 3% of nAChRs in mouse cortex (Mao et al., 2008) and found in only ∼10% of cortical interneurons (Figure 2)—but are increasingly viewed as functionally important. Unlike most nAChRs, α2 nAChRs exhibit little or no desensitization in the presence of agonist (Intskirveli et al., 2024; Jia et al., 2009) and therefore may be disproportionately active following systemic nicotine administration that produces effects lasting tens of minutes in mice (Intskirveli and Metherate, 2012; Kawai et al., 2011). Interneurons with α2 nAChRs are overwhelmingly (>90%) layer 5 Martinotti cells (Hilscher et al., 2017; Hostetler et al., 2023; Wu et al., 2023), although our infragranular group includes some cells in layer 6 (Intskirveli et al., 2024). These α2-MCs have axon projections that terminate in upper layers where they contact pyramidal cell apical dendrites and interneurons that, in turn, contact pyramidal cells (Donato et al., 2023). Thus, α2-MCs can inhibit and disinhibit, respectively, inputs to distal and proximal apical dendrites to shift the mode of cortical processing to favor thalamic input over cortico-cortical inputs (Intskirveli et al., 2024). Studies have shown that α2 nAChRs contribute critically to cortical functions including spectral integration in auditory cortex (Intskirveli et al., 2024), sensorimotor integration in motor cortex (Velica et al., 2025), reactivation of developmental plasticity in adult visual cortex (Sadahiro et al., 2020), and synchronization of neural oscillations (Hilscher et al., 2017). Notably, while rare in rodent cortex, α2 nAChRs are highly expressed in primate cortex at levels close to those of the predominant α4β2 nAChR, a striking species difference potentially related to enhanced cortical function in primates (Han et al., 2000).

The present study is the first description of age-related changes in α2 cells. The laminar distribution of α2 cells in young adult mice is entirely consistent with results from a different transgenic line (Chrna2-Cre mouse) (Hilscher et al., 2017). While overall numbers don’t change with age (Figure 2), there was a small increase in supragranular α2 cells and decrease in infragranular α2-MCs (Figure 1). In young adult mice, 86% of α2 cells co-expressed SOM, similar to the 83% in one study using the Chrna2-Cre mouse (Tokarska and Silberberg, 2022); however, another study in Chrna2-Cre mice reported only 30% co-label with SOM (Hilscher et al., 2017). Over the adult lifetime of FVB mice, the percentage of α2 cells co-labeled with SOM decreased progressively from 86 to 20% (Figure 3C). Since total α2 cells did not change over this time and total SOM cells decreased (Figure 2), this result implies that some α2 cells lose co-expression of SOM with increasing age. Finally, the 11% of α2 cells in young mice that were not co-labeled with either SOM or VIP (Figure 3B) may be non-VIP 5-HT3A receptor-expressing interneurons (Rudy et al., 2010), which were not examined in this study.

### 4.3 Chronic nicotine exposure and reversal of age-related decline of interneuron marker proteins

The most striking finding of this study is that 3-month CNE increased the numbers of interneurons with PV and VIP at three older ages to similar levels as in young adults (Figure 4). CNE increased SOM neurons only at 12 months and did not change α2 cell numbers. CNE overtly increased interneuron counts, rather than preventing the age-related decline, since the increase was observed even when CNE began after substantial decline had occurred. The age-related decline and CNE-induced increase in interneuron counts likely reflect altered expression of marker proteins, rather than loss and gain of neurons *per se* (Martin del Campo et al., 2012). In support of this notion, we found that CNE systematically increased intensities for PV fluorescence in 12-month-old mice (Figure 5B), suggesting increased expression of PV protein. Similarly, prior studies report age-related decreases in interneuron marker proteins without a loss of interneurons (de Villers-Sidani et al., 2010; Ouellet and de Villers-Sidani, 2014), and/or age-related decreases in the intensity of PV fluorescence (de Villers-Sidani et al., 2010; Ueno et al., 2018). Notably, behavioral training in older animals partly reverses the age-related decrease in PV fluorescence intensity, reducing the number of cells with weak fluorescence and increasing cells with strong fluorescence (de Villers-Sidani et al., 2010). That is, the effect of behavioral training resembles the effect of CNE in the present study. Our data showing that CNE increased interneuron counts to similar levels as in young adults (Figure 4), and increased PV fluorescence without exceeding the maximum intensity in controls (Figure 5B) may indicate that the levels of PV expression in young adult mice cannot be exceeded following CNE in older mice. Together, these findings suggest that the CNE-induced increases in interneuron numbers reflect increased expression of marker proteins in existing interneurons.

The mechanism by which CNE increased marker expression in the aging brain is not known, but a possible link is suggested by studies of nicotine’s putative anti-aging effects. Intriguingly, such effects—which include anti-inflammatory and neuroprotective actions (Majdi et al., 2018)—may occur via direct stimulation of metabolic pathways (Yang et al., 2023). These pathways are ubiquitous across organisms, decline with age, and are targeted by anti-aging treatments such as nicotinamide (niacin) supplements (Lautrup et al., 2019; Yoshino et al., 2018). Since the effects of nicotine on these metabolic pathways may be direct, i.e., independent of nAChRs, they could affect a very broad range of cellular processes (Yang et al., 2023). Future studies should explore the interrelationships among CNE, cell metabolism, and neural function in the aging brain.

## Data Availability

The original contributions presented in the study are included in the article/supplementary material, further inquiries can be directed to the corresponding author.

## References

[B1] Anderson L. Christianson G. Linden J. (2009). Mouse auditory cortex differs from visual and somatosensory cortices in the laminar distribution of cytochrome oxidase and acetylcholinesterase. *Brain Res.* 1252 130–142. 10.1016/j.brainres.2008.11.037 19061871

[B2] Araujo D. Lapchak P. Meaney M. Collier B. Quirion R. (1990). Effects of aging on nicotinic and muscarinic autoreceptor function in the rat brain: relationship to presynaptic cholinergic markers and binding sites. *J. Neurosci.* 10 3069–3078. 10.1523/JNEUROSCI.10-09-03069.1990 2398371 PMC6570246

[B3] Askew C. Intskirveli I. Metherate R. (2017). Systemic nicotine increases gain and narrows receptive fields in A1 via integrated cortical and subcortical actions. *eNeuro* 4:ENEURO.0192-17.2017. 10.1523/ENEURO.0192-17.2017 28660244 PMC5480142

[B4] Askew C. Lopez A. Wood M. Metherate R. (2019). Nicotine excites VIP interneurons to disinhibit pyramidal neurons in auditory cortex. *Synapse* 73:e22116. 10.1002/syn.22116 31081950 PMC6767604

[B5] Benowitz N. (1996). Cotinine as a biomarker of environmental tobacco smoke exposure. *Epidemiol. Rev.* 18 188–204. 10.1093/oxfordjournals.epirev.a017925 9021312

[B6] Bishop R. Qureshi F. Yan J. (2022). Age-related changes in neuronal receptive fields of primary auditory cortex in frequency, amplitude, and temporal domains. *Hear. Res.* 420:108504. 10.1016/j.heares.2022.108504 35421776

[B7] Brewton D. Kokash J. Jimenez O. Pena E. Razak K. (2016). Age-related deterioration of perineuronal nets in the primary auditory cortex of mice. *Front Aging Neurosci.* 8:270. 10.3389/fnagi.2016.00270 27877127 PMC5099154

[B8] Caspary D. Ling L. Turner J. Hughes L. (2008). Inhibitory neurotransmission, plasticity and aging in the mammalian central auditory system. *J. Exp. Biol.* 211 1781–1791. 10.1242/jeb.013581 18490394 PMC2409121

[B9] Cruikshank S. Killackey H. Metherate R. (2001). Parvalbumin and calbindin are differentially distributed within primary and secondary subregions of the mouse auditory forebrain. *Neuroscience* 105 553–569. 10.1016/s0306-4522(01)00226-3 11516823

[B10] Cruikshank S. Rose H. Metherate R. (2002). Auditory thalamocortical synaptic transmission in vitro. *J. Neurophysiol.* 87 361–384. 10.1152/jn.00549.2001 11784756

[B11] Dalton D. Cruickshanks K. Klein B. Klein R. Wiley T. Nondahl D. (2003). The impact of hearing loss on quality of life in older adults. *Gerontologist* 43 661–668. 10.1093/geront/43.5.661 14570962

[B12] de Villers-Sidani E. Alzghoul L. Zhou X. Simpson K. Lin R. Merzenich M. (2010). Recovery of functional and structural age-related changes in the rat primary auditory cortex with operant training. *Proc. Natl. Acad. Sci. U S A.* 107 13900–13905. 10.1073/pnas.1007885107 20643928 PMC2922281

[B13] Donato C. Balduino Victorino D. Cabezas C. Aguirre A. Lourenço J. Potier M. et al. (2023). Pharmacological signature and target specificity of inhibitory circuits formed by martinotti cells in the mouse barrel cortex. *J. Neurosci.* 43 14–27. 10.1523/JNEUROSCI.1661-21.2022 36384682 PMC9838699

[B14] Ghimire M. Cai R. Ling L. Hackett T. Caspary D. (2020). Nicotinic receptor subunit distribution in auditory cortex: Impact of aging on receptor number and function. *J. Neurosci.* 40 5724–5739. 10.1523/JNEUROSCI.0093-20.2020 32541068 PMC7380976

[B15] Gordon-Salant S. Yeni-Komshian G. Fitzgibbons P. Barrett J. (2006). Age-related differences in identification and discrimination of temporal cues in speech segments. *J. Acoust. Soc. Am.* 119 2455–2466. 10.1121/1.2171527 16642858

[B16] Han Z. Le Novère N. Zoli M. Hill J. Champtiaux N. Changeux J. (2000). Localization of nAChR subunit mRNAs in the brain of *Macaca mulatta*. *Eur. J. Neurosci.* 12 3664–3674. 10.1046/j.1460-9568.2000.00262.x 11029636

[B17] Hilscher M. Leão R. Edwards S. Leão K. Kullander K. (2017). Chrna2-martinotti cells synchronize layer 5 type A pyramidal cells via rebound excitation. *PLoS Biol.* 15:e2001392. 10.1371/journal.pbio.2001392 28182735 PMC5300109

[B18] Hilscher M. Mikulovic S. Perry S. Lundberg S. Kullander K. (2023). The alpha2 nicotinic acetylcholine receptor, a subunit with unique and selective expression in inhibitory interneurons associated with principal cells. *Pharmacol. Res.* 196:106895. 10.1016/j.phrs.2023.106895 37652281

[B19] Ho M. Li X. Wang J. Ohmen J. Friedman R. A. (2014). FVB/NJ mice demonstrate a youthful sensitivity to noise-induced hearing loss and provide a useful genetic model for the study of neural hearing loss. *Audiol. Neurotol. Extra* 4 1–11. 10.1159/000357770 24707282 PMC3972069

[B20] Hostetler R. Hu H. Agmon A. (2023). Genetically defined subtypes of somatostatin-containing cortical interneurons. *eNeuro* 10:ENEURO.0204-23.2023. 10.1523/ENEURO.0204-23.2023 37463742 PMC10414551

[B21] Hussain A. Erickson M. Rumschlag J. Razak K. (2026). Acute nicotine affects auditory brainstem responses in a frequency-, age-, and sex-dependent manner in mice. *Hear. Res.* 475:109619. 10.1016/j.heares.2026.109619 41905335

[B22] Intskirveli I. Metherate R. (2012). Nicotinic neuromodulation in auditory cortex requires MAPK activation in thalamocortical and intracortical circuits. *J. Neurophysiol.* 107 2782–2793. 10.1152/jn.01129.2011 22357798 PMC3362282

[B23] Intskirveli I. Gil S. Lazar R. Metherate R. (2024). Alpha-2 nicotinic acetylcholine receptors regulate spectral integration in auditory cortex. *Front. Neural Circuits* 18:1492452. 10.3389/fncir.2024.1492452 39553292 PMC11563825

[B24] Jia Y. Yamazaki Y. Nakauchi S. Sumikawa K. (2009). Alpha2 nicotine receptors function as a molecular switch to continuously excite a subset of interneurons in rat hippocampal circuits. *Eur. J. Neurosci.* 29 1588–1603. 10.1111/j.1460-9568.2009.06706.x 19385992 PMC2915898

[B25] Jones S. Yakel J. (1997). Functional nicotinic ACh receptors on interneurones in the rat hippocampus. *J. Physiol.* 504 603–610. 10.1111/j.1469-7793.1997.603bd.x 9401968 PMC1159964

[B26] Kawai H. Kang H. Metherate R. (2011). Heightened nicotinic regulation of auditory cortex during adolescence. *J. Neurosci.* 31 14367–14377. 10.1523/JNEUROSCI.1705-11.2011 21976522 PMC3210642

[B27] Kawai H. Lazar R. Metherate R. (2007). Nicotinic control of axon excitability regulates thalamocortical transmission. *Nat. Neurosci.* 10 1168–1175. 10.1038/nn1956 17704774

[B28] Lakatos P. Musacchia G. O’Connel M. Falchier A. Javitt D. Schroeder C. (2013). The spectrotemporal filter mechanism of auditory selective attention. *Neuron* 77 750–761. 10.1016/j.neuron.2012.11.034 23439126 PMC3583016

[B29] Lautrup S. Sinclair D. Mattson M. Fang E. F. (2019). NAD+ in brain aging and neurodegenerative disorders. *Cell. Metab.* 30 630–655. 10.1016/j.cmet.2019.09.001 31577933 PMC6787556

[B30] Liang K. Poytress B. Weinberger N. Metherate R. (2008). Nicotinic modulation of tone-evoked responses in auditory cortex reflects the strength of prior auditory learning. *Neurobiol. Learn. Mem.* 90 138–146. 10.1016/j.nlm.2008.02.006 18378471 PMC2464281

[B31] Lotfipour S. Byun J. Leach P. Fowler C. Murphy N. Kenny P. et al. (2013). Targeted deletion of the mouse α2 nicotinic acetylcholine receptor subunit gene (Chrna2) potentiates nicotine-modulated behaviors. *J. Neurosci.* 33 7728–7741. 10.1523/JNEUROSCI.4731-12.2013 23637165 PMC3831006

[B32] Lotfipour S. Mojica C. Nakauchi S. Lipovsek M. Silverstein S. Cushman J. et al. (2017). α2* Nicotinic acetylcholine receptors influence hippocampus-dependent learning and memory in adolescent mice. *Learn. Mem.* 24 231–244. 10.1101/lm.045369.117 28507032 PMC5435881

[B33] Majdi A. Sadigh-Eteghad S. Gjedde A. (2021). Effects of transdermal nicotine delivery on cognitive outcomes: A meta-analysis. *Acta Neurol. Scand.* 144 179–191. 10.1111/ane.13436 33899218

[B34] Majdi A. Sadigh-Eteghad S. Talebi M. Farajdokht F. Erfani M. Mahmoudi J. et al. (2018). Nicotine modulates cognitive function in D-galactose-induced senescence in mice. *Front. Aging Neurosci.* 10:194. 10.3389/fnagi.2018.00194 30061821 PMC6055060

[B35] Mao D. Perry D. Yasuda R. Wolfe B. Kellar K. (2008). The alpha4beta2alpha5 nicotinic cholinergic receptor in rat brain is resistant to up-regulation by nicotine in vivo. *J. Neurochem.* 104 446–456. 10.1111/j.1471-4159.2007.05011.x 17961152

[B36] Martin del Campo H. N. Measor K. R. Razak K. A. (2012). Parvalbumin immunoreactivity in the auditory cortex of a mouse model of presbycusis. *Hear. Res.* 294 31–39. 10.1016/j.heares.2012.08.017 23010334

[B37] Mineur Y. Ernstsen C. Islam A. Lefoli Maibom K. Picciotto M. (2020). Hippocampal knockdown of α2 nicotinic or M1 muscarinic acetylcholine receptors in C57BL/6J male mice impairs cued fear conditioning. *Genes Brain Behav*. 19:e12677. 10.1111/gbb.12677 32447811 PMC8018799

[B38] Newhouse P. Kellar K. Aisen P. White H. Wesnes K. Coderre E. et al. (2012). Nicotine treatment of mild cognitive impairment: A 6-month double-blind pilot clinical trial. *Neurology* 78 91–101. 10.1212/WNL.0b013e31823efcbb 22232050 PMC3466669

[B39] O’Connell M. Barczak A. Schroeder C. Lakatos P. (2014). Layer specific sharpening of frequency tuning by selective attention in primary auditory cortex. *J. Neurosci.* 34 16496–16508. 10.1523/JNEUROSCI.2055-14.2014 25471586 PMC4252556

[B40] Okamoto H. Stracke H. Wolters C. Schmael F. Pantev C. (2007). Attention improves population-level frequency tuning in human auditory cortex. *J. Neurosci.* 27 10383–10390. 10.1523/JNEUROSCI.2963-07.2007 17898210 PMC6673146

[B41] Oliveira V. Carrara R. Simoes D. Saggioro F. Carlotti C. Covas D. et al. (2010). Sudan Black B treatment reduces autofluorescence and improves resolution of in situ hybridization specific fluorescent signals of brain sections. *Histol. Histopathol.* 25 1017–1024. 10.14670/HH-25.1017 20552552

[B42] Ouda L. Druga R. Syka J. (2008). Changes in parvalbumin immunoreactivity with aging in the central auditory system of the rat. *Exp. Gerontol.* 43 782–789. 10.1016/j.exger.2008.04.001 18486384

[B43] Ouellet L. de Villers-Sidani E. (2014). Trajectory of the main GABAergic interneuron populations from early development to old age in the rat primary auditory cortex. *Front. Neuroanat.* 8:40. 10.3389/fnana.2014.00040 24917792 PMC4040493

[B44] Reinhard S. Abundez-Toledo M. Espinoza K. Razak K. (2019). Effects of developmental noise exposure on inhibitory cell densities and perineuronal nets in A1 and AAF of mice. *Hear. Res.* 381:107781. 10.1016/j.heares.2019.107781 31425896

[B45] Rogalla M. Hildebrandt K. (2020). Aging but not age-related hearing loss dominates the decrease of parvalbumin immunoreactivity in the primary auditory cortex of mice. *eNeuro* 7:ENEURO.0511-19.2020. 10.1523/ENEURO.0511-19.2020 32327469 PMC7210488

[B46] Rogers S. Gahring L. Collins A. Marks M. (1998). Age-related changes in neuronal nicotinic acetylcholine receptor subunit alpha4 expression are modified by long-term nicotine administration. *J. Neurosci.* 18 4825–4832. 10.1523/JNEUROSCI.18-13-04825.1998 9634548 PMC6792565

[B47] Rudy B. Fishell G. Lee S. Hjerling-Leffler J. (2010). Three groups of interneurons account for nearly 100% of neocortical GABAergic neurons. *Dev. Neurobiol.* 71 45–61. 10.1002/dneu.20853 21154909 PMC3556905

[B48] Rumschlag J. Razak K. (2021). Age-related changes in event related potentials, steady state responses and temporal processing in the auditory cortex of mice with severe or mild hearing loss. *Hear. Res.* 412:108380. 10.1016/j.heares.2021.108380 34758398

[B49] Rumschlag J. Lovelace J. Kokash J. Hussain A. Razak K. (2022). Nicotine reduces age-related changes in cortical neural oscillations without affecting auditory brainstem responses. *Neurobiol. Aging* 120 10–26. 10.1016/j.neurobiolaging.2022.07.014 36084545

[B50] Sadahiro M. Demars M. Burman P. Yevoo P. Zimmer A. Morishita H. (2020). Activation of somatostatin interneurons by nicotinic modulator Lypd6 enhances plasticity and functional recovery in the adult mouse visual cortex. *J. Neurosci.* 40 5214–5227. 10.1523/JNEUROSCI.1373-19.2020 32467358 PMC7329312

[B51] Shilling-Scrivo K. Mittelstadt J. Kanold P. O. (2021). Altered response dynamics and increased population correlation to tonal stimuli embedded in noise in aging auditory cortex. *J. Neurosci.* 41 9650–9668. 10.1523/JNEUROSCI.0839-21.2021 34611028 PMC8612470

[B52] Siwani S. Franca A. S. C. Mikulovic S. Reis A. Hilscher M. M. Edwards S. J. et al. (2018). OLMalpha2 cells bidirectionally modulate learning. *Neuron* 99 404–412.e403. 10.1016/j.neuron.2018.06.022 29983324

[B53] Slaker M. L. Harkness J. H. Sorg B. A. (2016). A standardized and automated method of perineuronal net analysis using Wisteria floribunda agglutinin staining intensity. *IBRO Rep.* 1 54–60. 10.1016/j.ibror.2016.10.001 28713865 PMC5507617

[B54] Sottile S. Y. Ling L. Cox B. C. Caspary D. M. (2017). Impact of ageing on postsynaptic neuronal nicotinic neurotransmission in auditory thalamus. *J. Physiol.* 595 5375–5385. 10.1113/JP274467 28585699 PMC5538226

[B55] Stewart K. Schroeder V. A. (2017). *JoVE Science Education. Blood Withdrawal II. JoVE.* Available online at: https://app.jove.com/v/10247/collecting-blood-from-facial-submandibular-saphenous-femoral-veins

[B56] Sun S. Kapolowicz M. R. Richardson M. Metherate R. Zeng F. G. (2021). Task-dependent effects of nicotine treatment on auditory performance in young-adult and elderly human nonsmokers. *Sci. Rep.* 11:13187. 10.1038/s41598-021-92588-z 34162968 PMC8222263

[B57] Tokarska A. Silberberg G. (2022). GABAergic interneurons expressing the alpha2 nicotinic receptor subunit are functionally integrated in the striatal microcircuit. *Cell. Rep.* 39:110842. 10.1016/j.celrep.2022.110842 35613598

[B58] Turner J. G. Hughes L. F. Caspary D. M. (2005). Affects of aging on receptive fields in rat primary auditory cortex layer V neurons. *J. Neurophysiol.* 94 2738–2747. 10.1152/jn.00362.2005 16000522

[B59] Ueno H. Takao K. Suemitsu S. Murakami S. Kitamura N. Wani K. et al. (2018). Age-dependent and region-specific alteration of parvalbumin neurons and perineuronal nets in the mouse cerebral cortex. *Neurochem. Int.* 112 59–70. 10.1016/j.neuint.2017.11.001 29126935

[B60] Velica A. Henriksson K. Malfatti T. Ciralli B. Nogueira I. Asimakidou E. et al. (2025). Layer-specific connectivity and functional interference of Chrna2+ layer 5 martinotti cells in the primary motor cortex. *Eur. J. Neurosci.* 61:e70086. 10.1111/ejn.70086 40170286 PMC11962176

[B61] Weinstein B. E. Ventry I. M. (1982). Hearing impairment and social isolation in the elderly. *J. Speech Hear. Res.* 25 593–599. 10.1044/jshr.2504.593 7162161

[B62] Whitehouse P. J. Martino A. M. Wagster M. V. Price D. L. Mayeux R. Atack J. R. et al. (1988). Reductions in [3H]nicotinic acetylcholine binding in Alzheimer’s disease and Parkinson’s disease: An autoradiographic study. *Neurology* 38 720–723. 10.1212/wnl.38.5.720 3362368

[B63] Wu S. J. Sevier E. Dwivedi D. Saldi G. A. Hairston A. Yu S. et al. (2023). Cortical somatostatin interneuron subtypes form cell-type-specific circuits. *Neuron* 111 2675–2692.e79. 10.1016/j.neuron.2023.05.032 37390821 PMC11645782

[B64] Yang L. Shen J. Liu C. Kuang Z. Tang Y. Qian Z. et al. (2023). Nicotine rebalances NAD(+) homeostasis and improves aging-related symptoms in male mice by enhancing NAMPT activity. *Nat. Commun.* 14:900. 10.1038/s41467-023-36543-8 36797299 PMC9935903

[B65] Yoshino J. Baur J. A. Imai S. I. (2018). NAD(+) intermediates: The Biology and therapeutic potential of NMN and NR. *Cell. Metab.* 27 513–528. 10.1016/j.cmet.2017.11.002 29249689 PMC5842119

[B66] Yuan R. Tsaih S. W. Petkova S. B. Marin de Evsikova C. Xing S. Marion M. A. et al. (2009). Aging in inbred strains of mice: Study design and interim report on median lifespans and circulating IGF1 levels. . *Aging Cell* 8 277–287. 10.1111/j.1474-9726.2009.00478.x 19627267 PMC2768517

[B67] Zhang X. Wahlstrom G. Nordberg A. (1990). Influence of development and aging on nicotinic receptor subtypes in rodent brain. *Int. J. Dev. Neurosci.* 8 715–721. 10.1016/0736-5748(90)90065-A 2288245

[B68] Zheng Q. Y. Johnson K. R. Erway L. C. (1999). Assessment of hearing in 80 inbred strains of mice by ABR threshold analyses. *Hear. Res.* 130 94–107. 10.1016/s0378-5955(99)00003-9 10320101 PMC2855304

